# Changes in the Laser-Processed Ti6Al4V Titanium Alloy Surface Observed by Using Raman Spectroscopy

**DOI:** 10.3390/ma15207153

**Published:** 2022-10-14

**Authors:** Mariusz Dudek, Zuzanna Wawryniuk, Malwina Nesteruk, Adam Rosowski, Michał Cichomski, Marek Kozicki, Robert Święcik

**Affiliations:** 1 Institute of Materials Science and Engineering, Lodz University of Technology, Stefanowskiego 1/15, 90-924 Lodz, Poland; 2 SPI Lasers, 3 Wellington Park, Tollbar Way, Hedge End, Southampton, Hampshire SO30 2QU, UK; 3 Institute for Manufacturing, University of Cambridge, 17 Charles Babbage Road, Cambridge CB3 0FS, UK; 4 Department of Materials Technology and Chemistry, Faculty of Chemistry, University of Lodz, Pomorska 163, 90-236 Lodz, Poland; 5 Department of Mechanical Engineering, Informatics and Chemistry of Polymer Materials, Faculty of Materials Technologies and Textile Design, Lodz University of Technology, Żeromskiego 116, 90-924 Lodz, Poland; 6 Institute of Machine Tools and Production Engineering, Lodz University of Technology, Stefanowskiego 1/15, 90-924 Lodz, Poland

**Keywords:** Ti6Al4V alloy, laser functionalization, surface complexity, Raman spectroscopy

## Abstract

This works reports on the effects of treating the surface of Ti6Al4V titanium alloy samples with a laser with a wavelength of 1064 nm, operating in a pulsed and continuous mode. The obtained surfaces with different roughness, complexity and wettability were examined by Raman spectroscopy in order to recognize the presence of titanium oxides on the functionalized surface. The layer of titanium oxides on the surface with the identified rutile phase obtained by laser treatment in the continuous wave mode is a reason for a hydrophobic surface that appeared 50 days after the treatment process. In the case of the surface obtained by the pulsed laser process, only local points at which the Raman bands attributed to the metastable phases anatase and brookite of TiO_2_ can be identified. In this treatment process, complete surface hydrophilicity was observed during 29 days after the functionalization process (maximal contact angle observed during this time was 68.4 deg). For some functionalization processes of different parameters, the contact angle remained immeasurable until 119 days after the functionalization process. In summary, Raman spectroscopy identifies surface changes of Ti6Al4V after laser processing.

## 1. Introduction

One of the most popular engineering materials in use today is the titanium alloy Ti6Al4V. It is widely used in various industries—aerospace, automotive, chemical plants, power generators, dentistry and medicine—due to its unique properties such as high strength-to-weight ratio, low density, biocompatibility and excellent corrosion resistance [1,2,3]. Nevertheless, intensive research is still carried out to improve the physicochemical properties of its surface, ranging from shot peening, thermal oxidation, and ion implantation to numerous coating techniques [4,5,6,7,8,9,10]. 

In recent decades, the use of lasers in the surface engineering and powder melting of various materials has grown in importance [11,12,13,14,15,16,17,18]. Laser treatment is also successfully used in the case of titanium alloys. Cunha et al. [19] showed that laser treatment significantly reduces *S. aureus* adhesion and biofilm formation compared to polished reference samples. Laser-induced texture nanotopography reduces bacterial adhesion, since the size of individual features and the average distance between them inhibit the penetration of bacteria, thus reducing the area of contact between a single bacterium and a metal. Dinh et al. [20] developed a method for producing a superhydrophobic surface on titanium. Kummel et al. [21] used laser texturing to create linear channel textures to improve the poor tribological properties of Ti6Al4V. Li et al. [22] obtained an antireflection microstructure with superhydrophobic properties, using a nanosecond laser treatment system to treat the surface of the titanium alloy. Grabowski et al. [23] demonstrated that the appropriate selection of the pulse laser energy density and duration allows the thin layer of the Ti6Al4V surface to be melted. Moreover, Liu et al. [24] achieved bionic, self-cleaning and anti-icing Ti6Al4V alloy surfaces.

In this study, the surface of a Ti6Al4V titanium alloy sample was treated with a laser with a wavelength of 1064 nm, operating in the pulse and continuous wave mode. The obtained surfaces were analyzed using optical and confocal microscopes, and a contact angle measurement technique. The Raman spectroscopy technique was then used to recognize the presence of titanium oxides on the functionalized surface to explain their wettability.

## 2. Materials and Methods

### 2.1. Materials

Titanium alloy Ti6Al4V samples (5 mm thick and 1 inch diameter discs, MEDGAL Orthopedic Implants & Instruments, Ksieżyno, Poland) were grinded on 80 to 2000 grit sandpaper and polished by using a colloidal silica suspension with an automatic polisher machine MECATECH 334 (Presi, Paris, France)—surface roughness, *Ra* = 0.18 ± 0.03 µm. The polished samples were ultrasonically cleaned in an acetone bath.

### 2.2. Functionalisation

The sample functionalization process was carried out using a 20 W G3 SPI laser (SPI Lasers UK Ltd., Southampton, UK) with a wavelength of 1064 nm. During the functionalization process, the laser was operated in a continuous mode and pulse mode with a frequency of 35 kHz and a pulse duration of 220 ns. The laser beam was delivered to the 2-axis XLR8 scanning head and the Linos F-Theta 160 mm focusing lens (the diameter of the laser spot on the substrate surface was about 40 µm). The scanner head with the F-Theta lens made it possible to move the focused beam across the substrate while maintaining the focus position on the surface of the substrate. The 16 × 16 mm^2^ scan area was processed with different scan speed and hatch distance as shown in Table 1. In the case of pulsed laser treatment of the titanium alloy surface, the scanning speed was selected to obtain separate craters after the ablation process and with minimal surface overlapping by craters. In order to observe changes in the surface topography after working with the laser in a continuous mode, the scanning speed was reduced to 75 mm/s (the laser power absorption was significantly increased [17]). No purge gas was applied to the laser processing of the titanium alloy samples.

### 2.3. Morphology of Samples

The surface of the titanium alloy samples after laser treatment was examined using optical and confocal microscopy. A confocal laser scanning microscope (CLSM, Nikon MA200, Nikon, Tokyo, Japan), was used to obtain maps of the treated surfaces. A Nikon MA200 microscope (as a metallographic microscope) was also used to take photos of the surface. The surface maps obtained by CLSM were analyzed using MountainsLab^®^ Premium software package (Digital Surf, Besançon, France) to determine the diameter and depth of created craters, roughness and complexity (the ratio of actual surface of the sample to its geometrical dimensions) of the treated surfaces.

### 2.4. Contact Angle Measurements

The surface wettability was measured in a laboratory atmosphere at a relative humidity of 45 ± 5% and a temperature of 22 ± 2 °C using the DSA-25 drop shape analysis system (KRÜSS GmbH, Hamburg, Germany). Two liquids were used to measure the contact angle, namely deionized water (POCH SA, Gliwice, Poland) and diiodomethane droplets (POCH SA, Gliwice, Poland). The drops of 0.8 µL volume were applied to the surfaces with an automatic syringe. The static contact angle values are given for five different droplets deposited on each sample.

### 2.5. Raman Spectroscopy Measurements

Raman spectroscopy measurements were carried out using a Renishaw inVia Raman microscope (Renishaw, Wotton-under-Edge, UK) equipped with a 532 nm laser set up in a backscatter geometry (objective ×20) with applied wavenumbers ranging from 100 to 3200 cm^−1^. Spectral profiles were collected at a laser power of 2.1 mW, an integration time of 100 s and a length of about 100 µm at 4 µm intervals. At selected points, the spectrum was collected once. All measurements were performed at room temperature and in an air atmosphere. Further analysis of Raman spectra was preceded by the removal of cosmic rays and subtraction of the baseline.

## 3. Results and Discussion

### 3.1. Morphology of Samples

**Figure 1:**
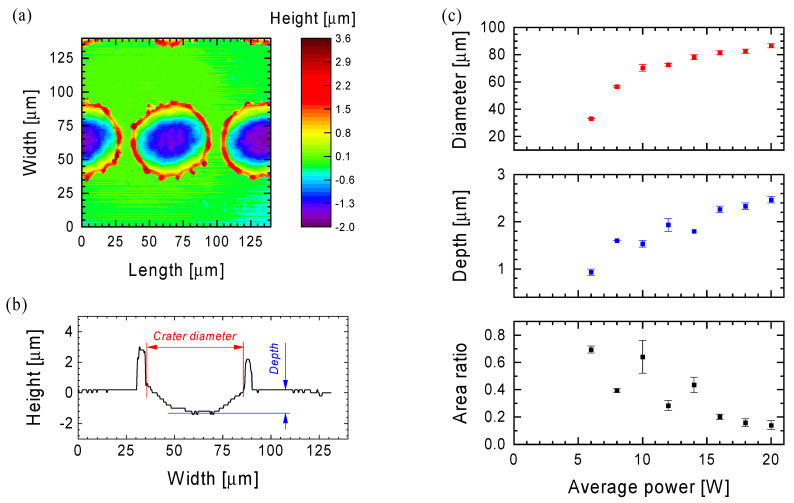
Surface characteristics of the Ti6Al4V alloy obtained from the confocal microscope measurements (magnification ×100), after the functionalization process carried out at the frequency of 35 kHz, the hatch distance of 100 µm and the scanning speed of 2400 mm/s. Example of a 2D crater map (**a**) and a selected crater profile (**b**) across the laser beam scanning line with the method of estimating the crater diameter and depth, after laser treatment at an average power of 8 W. (**c**) The crater diameter, depth and field ratio of the crater surface to the displaced sample material above its surface as a function of the average laser power.

The results of laser processing of various materials depend on parameters related to the laser used (wavelength, average power, frequency and duration of the pulse) and the scanning head (F-Theta lens, scanning speed and hatch distance). In order to demonstrate the influence of average power on the created crater on the surface of the Ti6Al4V titanium alloy by means of a laser pulse repeated with a frequency of 35 kHz (pulse duration 220 ns), a scanning speed of 2400 mm/s and a hatch distance of 100 µm were selected. Figure 1a shows an example of a 2D surface map with craters obtained from confocal microscope measurements (×100 magnification of the microscope objective), on the basis of which the crater profile across the laser beam scanning line was selected (Figure 1b) and analyzed. 

The data obtained confirm previous observations from an optical microscope (in particular in the dark field—not shown here) that two crater diameters are identified at the surface level: the lower one describing the actual crater below the surface of the sample and the upper one describing the crater with the sample material displaced above their surface. Figure 1c summarizes the results of estimating the inner diameter of the crater and its depth as a function of the average laser power used in the process of functionalization of the titanium alloy surface. Additionally, in Figure 1c the ratio of the crater surface area to the displaced sample material above their surface is shown. As expected, the diameter and depth of the crater increase as the average laser power increases. It should be emphasized here that in the case of the two lowest applied powers, the surface maps made did not allow for the identification of profiles and their further characterization (the craters were not visible). Contrary to the diameter and depth of the crater, the estimated surface ratio decreases with the laser power. This indicates that with selected laser parameters there is a power threshold (~5 W) below which the surface of the titanium alloy is not modified (denotes: macroscopic level; not molecular level), and after exceeding a certain power value (~12 W), the ablation process is the dominant process of surface functionalization in relation to mass displacement from the crater volume out of the area of operation of the laser beam.

**Figure 2:**
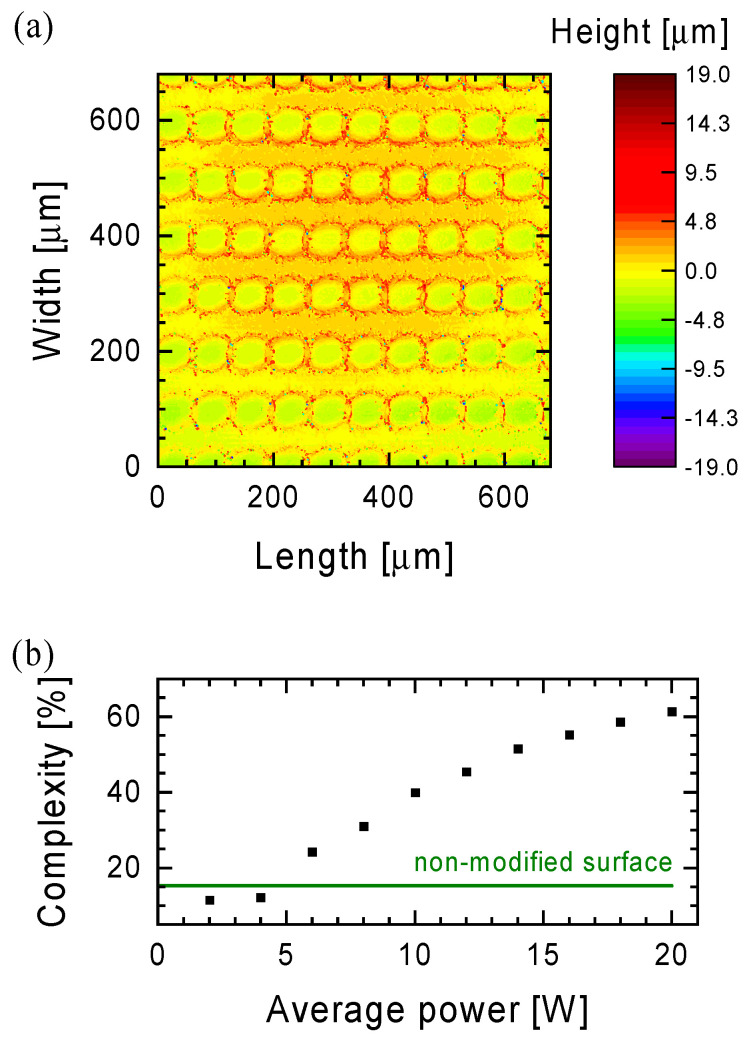
An example of a 2D map of craters on the surface of the Ti6Al4V alloy after the functionalization process at an average laser power of 12 W obtained by measurements with a confocal microscope at ×20 magnification (**a**). The results of the complexity estimation as a function of the average laser power used in the process of surface functionalization of the Ti6Al4V alloy (**b**). The functionalization process was carried out at a frequency of 35 kHz, a hatch distance of 100 µm and a scanning speed of 2400 mm/s. The green arrow in the graph (**b**) denotes the value for non-modified surface of the alloy.

An example of 2D maps from Figure 1a (×100 objective magnification) and Figure 2a (×20 objective magnification) shows that roughness is not a reliable parameter for comparing the surfaces produced by the laser. Depending on the parameters of the laser treatment, next to the areas exposed to the laser beam, there are also areas that have not been exposed to it. In order to overcome this problem, the complexity of the Ti6Al4V substrate was chosen as the parameter for the comparison of the laser treatment properties, which describes how much larger is the actual surface of the sample than its geometrical dimensions:

(1) $$complexity=\left(\frac{\mathrm{actual}\;\mathrm{surface}\;}{\mathrm{geometrical}\;\mathrm{dimensions}}-1\right)\cdot 100\%,$$

The complexity analysis was carried out for 2D maps obtained at ×20 magnification of the confocal microscope (Figure 2a), and the results are shown (Figure 2b) as a function of an average laser power for the process carried out at 35 kHz frequency, 100 µm hatch distance and 2400 mm/s scan speed. For selected parameters of the functionalization process (frequency 35 kHz, hatch distance 100 µm and scanning speed 2400 mm/s), the complexity of the surface increases with the increase in the average laser power. The same phenomenon was observed for the diameter and depth of the crater (Figure 1c). The results of the surface complexity assessment also confirm that below ~5 W of the average laser power, the effect of the laser beam is invisible. The estimated surface complexity values are lower compared to the Ti6Al4V surface after polishing, which was equal to 15.2% (the green arrow in Figure 2b). The reason for this observation is a difference in sample preparation prior to the laser functionalization process. Summarizing the discussed case of the functionalization process at a frequency of 35 kHz, a hatch distance of 100 µm and a scanning speed of 2400 mm/s, the highest value of the surface complexity (61.5%) was obtained with an average laser power of 20 W.

**Figure 3:**
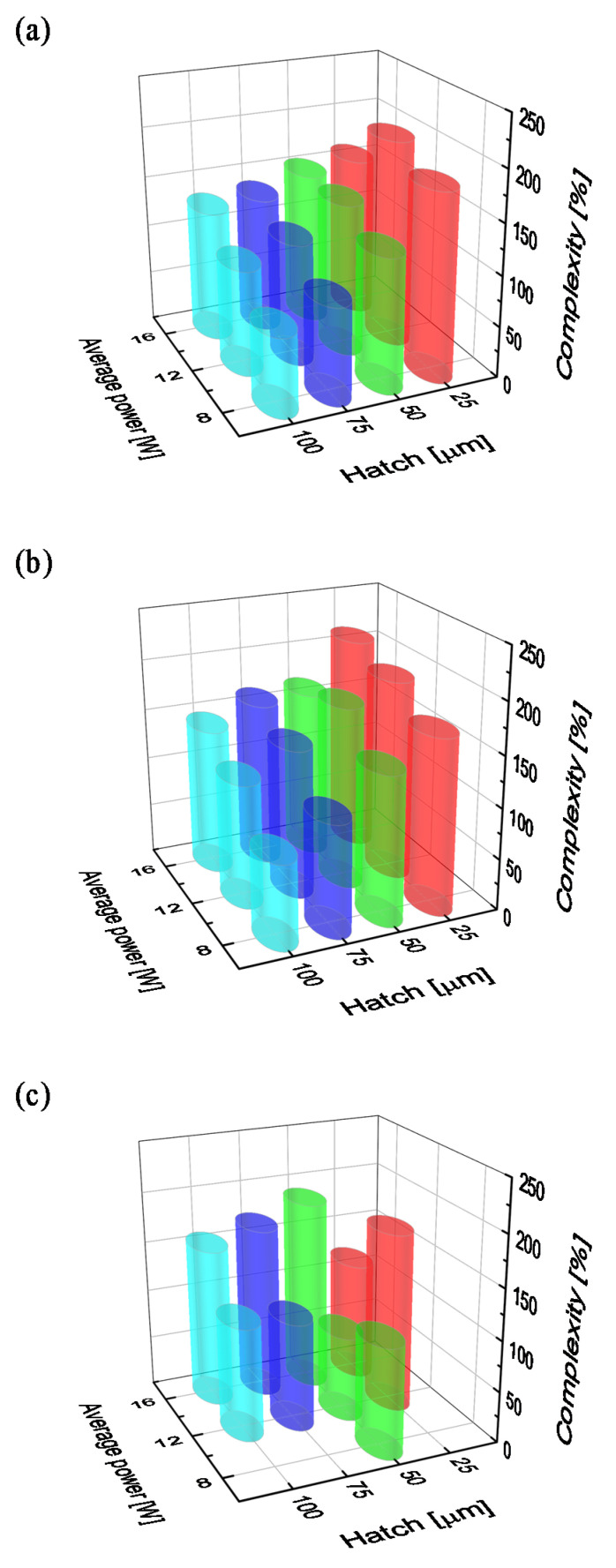
The surface complexity of the Ti6Al4V alloy as a function of the average laser power and hatch distance at the selected scanning speed during laser processing in pulse mode: 1800 mm/s (**a**) and 1200 mm/s (**b**), and at a continuous wave with a scanning speed of 75 mm/s (**c**).

**Figure 4:**
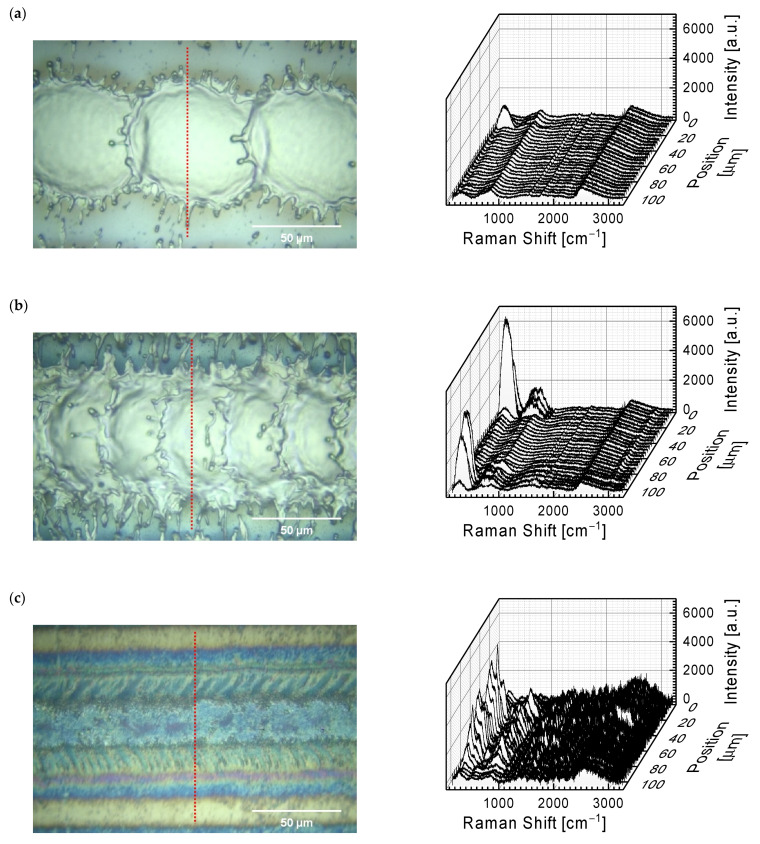
Raman spectra profiles measured after laser processing with an average power of 12 W and a hatch distance of 100 μm in the case of (**a**) pulse mode with a scanning speed of 2400 mm/s, (**b**) pulse mode with a scanning speed of 1200 mm/s, and (**c**) continuous wave with a scanning speed of 75 mm/s. In the photos, the dotted red line shows the place where the Raman spectra were collected (where 0 µm denotes the top of the line and 100 µm the bottom of the line) at 4 µm intervals.

The complexity of the surface is influenced not only by the average laser power, but also by the parameters of surface scanning with a laser, which include scanning speed and hatch distance. For the same frequency (35 kHz), the next functionalization process was carried out at the scanning speed of 1800 and 1200 mm/s (Figure 3a,b, respectively) and the hatch distance of 100, 75, 50 and 25 µm. Reducing the scanning speed from 2400 mm/s leads to an increase in the value of surface coverage with the laser beam (the next crater partially overlaps a previous one—see the photo in Figure 4b) and finally to an increase in the complexity of the surface. At 16 W average laser power and 100 µm hatch distance, the surface complexity is two-times higher (128.9% and 143.2% for 1800 and 1200 mm/s scanning speed, respectively), and it reaches the maximal value (61.5%) in the case of the treatment process with 2400 mm/s scanning speed. A further increase in surface complexity for a constant scanning speed can be obtained by reducing the hatch distance. Thus, for a hatch distance of 25 µm, a surface complexity of about 200% can be achieved (Figure 3a,b).

As shown in Figure 3c, a very similar surface complexity to the one discussed above can also be obtained in the case of continuous laser operation. The question then arises, will the other surface properties be the same with a similar surface complexity? To check this, measurements were made of the wettability of the surfaces.

### 3.2. Hydrophilic-Lipophilic Nature of Samples

Measurements of the contact angle for water showed that during the few days after laser functionalization of the Ti6Al4V surface for selected parameters discussed above, the water droplets immediately spread out (contact angle for the laser untreated, clean Ti6Al4V surfaces was 42.4 ± 2.3 deg). The first measurable water drops appeared for the functionalization process with a continuous laser wave 2 days after the functionalization process. In the case of the pulse-mode process (35 kHz frequency), the first measurable water drops were observed 29 days after the functionalization process. Table 2 summarizes the results, the information when the drop of water does not spread after the functionalization process and the first measured value of the contact angle. It is worth noting that in several cases of the laser treatment parameters discussed, 119 days after its implementation, the contact angle was still not possible to be measured; the water droplet on the modified surface continued to spread out immediately after deposition.

After the first day, when during the measurements the drop did not spread after being applied to the surface, the value of the contact angle increased with time with subsequent measurements. In the case of processes carried out for continuous laser operation, in most cases, after 50 days from the functionalization process, the surface becomes hydrophobic, while in the case of treatments in the pulsed laser mode, the droplets spread out. This result is in contradiction with literature reports, in which for the functionalization processes of titanium alloys, using a pulsed laser, hydrophobic surfaces were obtained almost immediately [24,25].

Comparing the results of the contact angle measurement of water (Table 2) with the results of the surface complexity measurements presented in Figure 2 and Figure 3, it can be seen that in the case of modification processes carried out in the pulsed laser mode, as the complexity (> 100%) increased, the water droplet spread out on the surface over time. In some cases, the measurement of the contact angle could not be made 119 days after the modification of this surface. In the case of a surface treated in continuous mode with the same complexity values, the measurement of the contact angle was possible already 9 days after the modification process was carried out.

### 3.3. Raman Spectroscopy Study

In order to explain the phenomena in which different behavior of a water droplet is observed with almost the same surface complexity, measurements of Raman spectra for the functionalized surfaces were performed. As the measurements at selected points did not give an unequivocal answer, which happened with the surface after laser treatment, the Raman spectra profiles were measured perpendicular to the direction of the laser beam at a distance of about 100 μm at a distance of 4 μm from each other. Figure 4 shows an example of Raman profiles with photos of surfaces, where the place of measurements of the Raman profile has been marked.

**Figure 5:**
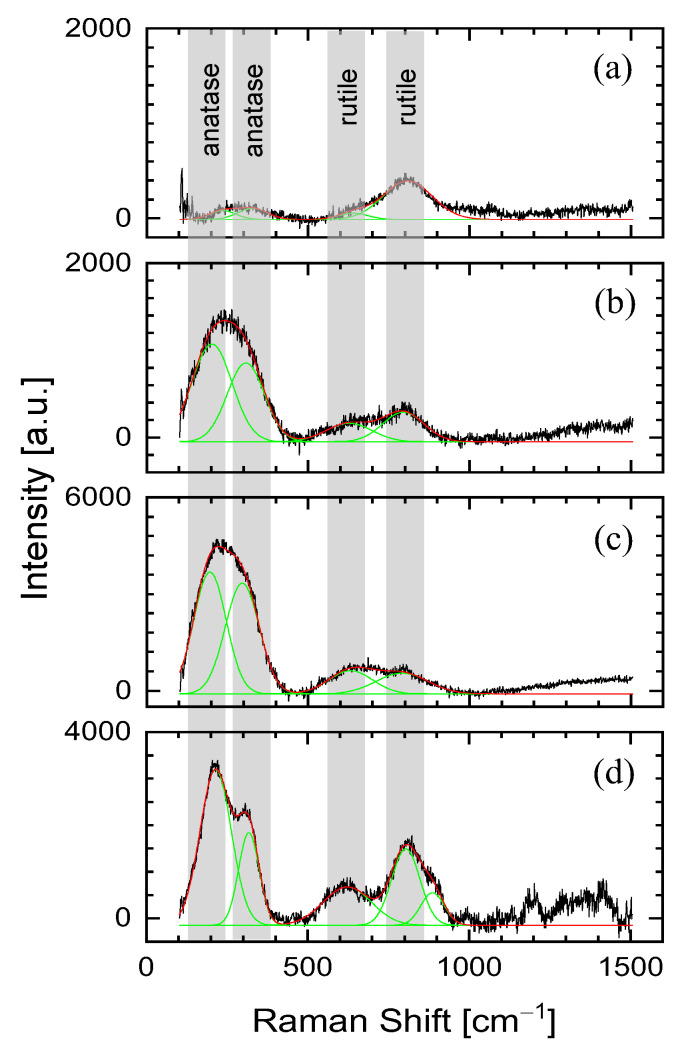
Examples of Raman spectra in the range 100–1500 cm^−1^ selected from the profiles presented in Figure 4: (**a**) an example of a spectrum for an untreated substrate made of Ti6Al4V alloy, (**b**) and (**c**) are for examples of spectra for the places with mass transferred from the crater as a result of the laser beam operation in pulse mode with a scanning speed of 2400 and 1200 mm/s, respectively, and (**d**) an example of a spectrum for a continuous wave treatment process. The modification process was carried out with an average laser power of 12 W.

Regardless of the process parameters, each of the profiles (Figure 4) shows a wide band from 2300 to 3000 cm^−1^ (with a maximum of ~2450 cm^−1^), characteristic for the metallic substrate. Moving towards the lower wavenumber (< 1000 cm^−1^), we can observe bands characteristic of metal oxides, especially titanium oxides. The Raman spectra profile for the Ti6Al4V surface after the functionalization process with a hatch distance of 100 µm and a scanning speed of 2400 mm/s at the laser beam repetition frequency of 35 kHz (Figure 4a) enables the observation of Raman spectra for the untreated surface and in the center of the crater. In both cases the spectra look the same—two broad bands are observed in the range of 100–500 cm^−1^ with a maximum of approx. 255 cm^−1^ and in the range of 500–1000 cm^−1^ with a maximum of approx. 800 cm^−1^. Figure 5 presents selected spectra from profiles in the range 100–1500 cm^−1^. These bands are the result of the presence of metal oxides on the tested surface, which are the effect of self-passivation of the titanium alloy, among others after the polishing process. It is worth noting that at the edge of the profile, and more precisely the Raman spectra, in the place where the photo shows the mass of the sample transferred from the crater as a result of the laser beam, the bands in the range of 100–500 cm^−1^ have a greater intensity. This indicates a higher level of oxidation of the tested material. This can be explained by the fact that the volume of molten titanium alloy was moved from the crater area. The increased temperature has a significant impact on the formation of titanium oxides in the process of its oxidation [26]. This is confirmed by the profile from Figure 4b for the functionalization process with the same average laser power and frequency, but twice lower scanning speed (1200 mm/s). The overlapping of the formed craters, as a result of greater surface coverage with the laser beam, leads to Raman spectra in the profile with greater intensity bands characteristic of metal oxides. It should be emphasized, however, that the discussed craters were made as a result of the ablation process, and therefore the Raman spectra are identical to those made for the non-laser treated surface after the polishing process.

Figure 4c shows a photo of a sample processed in a continuous laser mode and a profile of the Raman spectra. The photo shows that the surface of the sample after laser treatment looks like it has melted (this effect was obtained for a laser power of 12 W and a scanning speed of 75 mm/s). In the Raman spectra profile (at its edges), spectra typical for the untreated material can be observed; the hatch distance during the functionalization process was 100 µm. On the other hand, at the point where the laser beam passes the edges, sharp peaks typical for titanium oxides are visible in the bands below 1000 cm^−1^. In order to better describe this part of the spectra, Figure 5d is shown with one selected spectrum in the range 100–1500 cm^−1^. The selected spectrum was deconvoluted using Gaussian function and bands with maxima at 215 cm^−1^, 318 cm^−1^, 622 cm^−1^, 804 cm^−1^ and 887 cm^−1^ were identified. As shown in the spectra of Figure 5a–c, these bands can also be identified on the untreated and processed surface in a pulse mode, with one exception, a band at 887 cm^−1^.

The higher intensity of 622 cm^−1^ and 804 cm^−1^ in the case of the continuous wave functionalization process indicate that these two bands can be attributed to the rutile phase of TiO_2_ [27,28]. Nevertheless, bands at 215 cm^−1^ and 318 cm^−1^ can be attributed to the anatase and brookite phases of TiO_2_ [27,28,29]. The coexistence of these TiO_2_ phases on the surface of the functionalized Ti6Al4V alloy indicates that the analyzed laser treatment process allows to achieve local temperatures above 600 °C; the metastable anatase and brookite phases irreversibly convert into the equilibrium rutile phase upon heating above temperatures in the range of 600–800 °C [30]. 

Sharp bands in the Raman spectra for the processing of Ti6Al4V samples in the continuous laser mode indicate the formation of TiO_2_ layers on this surface. Their presence leads to a hydrophobic surface 50 days after the laser treatment process. The absence of continuous titanium oxide layers on the surface of the Ti6Al4V alloy, but the presence of point sites where bands associated with the metastable anatase and brookite phase of TiO_2_ can be identified, in the case of surfaces obtained in the pulsed laser mode, with the considered parameters of the laser process, leads to a complete hydrophilic surface during 29 days after the functionalization process (a drop of water on the modified surface continued to spread out). In a few functionalization process parameters, 119 days after the functionalization process, the contact angle was still not measurable.

## 4. Summary and Conclusions

The process of laser treatment of the Ti6Al4V alloy surface is a simple technique of surface functionalization. Depending on the laser and scanning parameters, this technique allows us to obtain surfaces with different roughness, complexity and wettability. Raman spectroscopy allows to recognize the presence of titanium oxides on the surface of a functionalized Ti6Al4V alloy in small (point) and larger (continuous) areas of this surface. Continuous wave laser treatment led to the formation of a functionalized layer of titanium oxides on the surface with an identified rutile phase. Their presence on the surface explains the measurable contact angle of the water drops a few days after the treatment (in one case, the maximum contact angle observed on the second day was about 64 deg, while for most cases it was unmeasurable at that time). After 50 days, in most of the cases considered for continuous wave processing, the surface became hydrophobic, while in the case of treatments in the pulsed laser mode, water droplets spread out. Raman spectroscopy measurements only show the presence of point sites where titanium oxide is identified in the metastable phases of anatase and brookite. There were places located on the modified surfaces where the transferred mass of the sample occurred from the craters as a result of the laser beam operation in the pulse mode. 

The presence of a layer of titanium oxides on the surface of the Ti6Al4V alloy with the identified rutile phase by Raman spectroscopy measurements, as a result of surface melting (and oxidation) in laser treatment in the continuous wave mode, the formation of surface hydrophobicity 50 days after the treatment.

Pulsed laser treatment leads to an increase in surface complexity as a result of the formation of craters and the partial transfer of mass (non-ablated) from them to the area in its vicinity. There are places where metastable phases of anatase and brookite phases of TiO_2_ can occur. Taking into account the parameters of the laser treatment, the first measurable contact angles for water drops (the maximum measured value is 68.4 deg) were observed on the 29th day after the functionalization process. However, in a few cases of the functionalization process, 119 days after its implementation, the contact angle was still not measurable; the water droplet on the modified surface was still spreading immediately after deposition.

## Figures and Tables

**Table 1:** Parameters of the laser system used in processing of titanium alloy samples.

Parameters	Pulse Mode (PM)	Continuous Wave (CW)
Scan speed [mm/s]	1200–2400	75
Hatch distance [µm]	25–100	25–100
Average power [W]	2–20	8–16

**Table 2:** The values of the contact angle [deg] measurements for water for selected parameters of Ti6Al4V functionalization. After a slash (/x), a day is given when the measurement of the contact angle was possible.

Average Laser Power [W]	Hatch Distance [µm]	Laser Mode and Scan Speed [mm/s]			
		PM 2400	PM 1800	PM 1200	CW 75
6	100	68.4 ± 1.3/29	-	-	-
8	25	-	32.5 ± 2.5/119	38.4 ± 2.8/35	-
8	50	-	49.1 ± 4.3/35	36.8 ± 4.5/55	64.3 ± 2.3/2
8	75	-	38.6 ± 3.8/35	50.6 ± 2.3/35	-
8	100	21.9 ± 1.6/29	34.3 ± 2.7/35	23.5 ± 1.8/35	-
10	100	26.7 ± 2.8/29	-	-	-
12	25	-	32.4 ± 1.5/55	17.6 ± 4.2/119	21.8 ± 2.3/4
12	50	-	48.3 ± 3.1/55	19.2 ± 2.5/55	36.4 ± 12.0/2
12	75	-	59.2 ± 3.6/55	72.9 ± 3.4/119	42.2 ± 5.8/2
12	100	22.4 ± 0.6/29	DWD	22.8 ± 2.3/55	49.5 ± 7.5/2
14	100	16.2 ± 1.9/29	-	-	-
16	25	-		DWD	23.8 ± 4.3/13
16	50	-		DWD	24.9 ± 1.8/9
16	75	-		DWD	17.6 ± 6.7/9
16	100	13.5 ± 1.7/29		DWD	16.1 ± 4.7/9
20	100	19.4 ± 0.5/29	-	-	-

DWD—spread out of water droplet on the modified surface 119 days after modification; CW—continuous mode of laser; PM—pulse mode of laser.

## Data Availability

The data supporting reported results is not stored in any publicly archived datasets. The readers can contact the corresponding author for any further clarification of the results obtained.
